# Master Protocols for Precision Medicine in Oncology: Overcoming Methodology of Randomized Clinical Trials

**DOI:** 10.3390/life11111253

**Published:** 2021-11-17

**Authors:** Raimondo Di Liello, Maria Carmela Piccirillo, Laura Arenare, Piera Gargiulo, Clorinda Schettino, Adriano Gravina, Francesco Perrone

**Affiliations:** 1 Oncologia Medica, Dipartimento di Medicina di Precisione, Università degli Studi della Campania “Luigi Vanvitelli”, Via S. Pansini 5, 80131 Napoli, Italy; raimondo.diliello@unicampania.it; 2 Unità Sperimentazioni Cliniche, Istituto Nazionale Tumori—IRCCS Fondazione G. Pascale, Via M. Semmola, 80131 Napoli, Italy; l.arenare@istitutotumori.na.it (L.A.); piera.gargiulo@istitutotumori.na.it (P.G.); c.schettino@istitutotumori.na.it (C.S.); a.gravina@istitutotumori.na.it (A.G.); f.perrone@istitutotumori.na.it (F.P.)

**Keywords:** personalized medicine, methodology, clinical trials, individualized treatment

## Abstract

Randomized clinical trials are considered the milestones of clinical research in oncology, and guided the development and approval of new compounds so far. In the last few years, however, molecular and genomic profiling led to a change of paradigm in therapeutic algorithms of many cancer types, with the spread of different biomarker-driven therapies (or targeted therapies). This scenario of “personalized medicine” revolutionized therapeutic strategies and the methodology of the supporting clinical research. New clinical trial designs are emerging to answer to the unmet clinical needs related to the development of these targeted therapies, overcoming the “classical” structure of randomized studies. Innovative trial designs able to evaluate more than one treatment in the same group of patients or many groups of patients with the same treatment (or both) are emerging as a possible future standard in clinical trial methodology. These are identified as “master protocols”, and include umbrella, basket and platform trials. In this review, we described the main characteristics of these new trial designs, focusing on the opportunities and limitations of their use in the era of personalized medicine.

## 1. Introduction

The methodology of clinical trials in oncology is continuously adapting to the rapid development of new targeted agents [1]. The spread of these biomarker-driven therapies led to a “rethinking” of the classic design of clinical trials, considering that the more the number of subgroups of patients with a specific molecular or genomic biomarker increases, the more the size of subgroups decreases [2].

In this scenario of “precision medicine”, the paradigm “one-size-fits-all” that characterized the cytotoxic agents’ era does not apply to the emergent clinical needs, and the “randomize-all” design seems no more suitable for the development of new drugs, in favor of trial designs structured to include different (but highly specific) small groups of patients to whom they offer the most personalized therapy [3]. Nevertheless, the time and costs of drug development also play a crucial role in this change of paradigm [4,5,6]. The known cost of genomic or molecular profiling must be added to the cost of planning and conducting different, parallel trials in each small patients’ subgroup, identified by a genomic/molecular alteration that is supposed to be targeted by the drug under evaluation [5]. The feasibility of such a strategy is also weakened by the difficulty of an efficient selection and enrollment process of subjects that fit all (usually many) protocol criteria without waste [7,8]. Moreover, designing different, parallel trials of the same drug for each specific cancer type, as according to the classic design of clinical trial, would lead to a number (resource and time-wasting) of new trials in the same cancer type, but with different molecular or genomic selection criteria, while having a number of new trials with the same molecular or genomic selection criteria, but in different cancer types. Therefore, to avoid conducting *n* different, parallel trials with *n* different compounds in *n* different biomarker subgroups in *n* different cancer types (moreover, often with the same comparison arm), clinical trials methodology proposed innovative trial designs that evaluate, within the same overall trial structure, more than one treatment in the same cancer type and its subgroup, or more than one subgroup with different treatments (or both) [9].

Such study designs are referred to as master protocols, defined as one unified study protocol that includes multiple sub-studies, able to efficiently answer multiple questions. Umbrella, basket and platform trials are included under this definition [10].

## 2. Umbrella Trials

**Figure 1:**
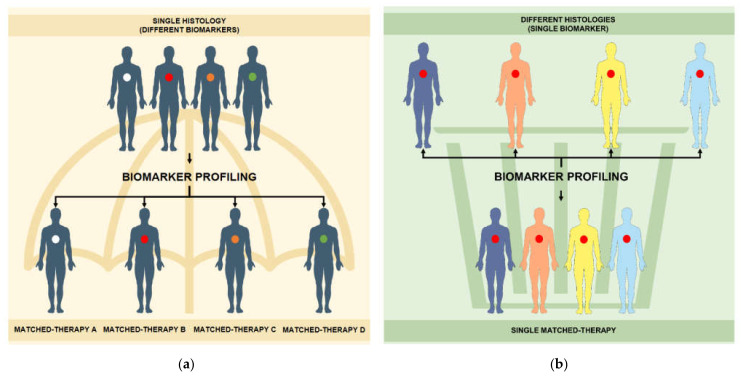
(**a**) Umbrella and (**b**) basket trial structure.

Umbrella design refers to prospective clinical trials that test multiple targeted compounds in a single cancer type, characterized by different known predictive biomarkers [11] (Figure 1a). In an umbrella trial, a general population selected by histology (e.g., lung cancer patients) is stratified in multiple, smaller subgroups assigned to specific biomarker-driven therapies. Umbrella design is applied often to cancer types where multiple biomarkers, with known (or supposed) predictive value, are present. Under an umbrella trial, multiple parallel sub-studies coexist, and every sub-study has its proper statistical parameters, based on the preclinical and clinical evidences available for the single match biomarker-targeted agent [9,12].

An example of umbrella trial is the VIKTORY (targeted agent eValuation In gastric cancer basket KORea, NCT02299648) study, which is designed to evaluate the best treatment for second line metastatic gastric cancer [13]. In this trial, patients were classified by genomic profiling in eight different biomarker groups (RAS aberration; TP53 mutation; PIK3CA mutation/amplification; MET amplification; MET protein overexpression; all negative; TSC2 deficient; or RICTOR amplification) and assigned to 10 independently designed phase 2 trials testing capivasertib (AKT inhibitor), savolitinib (MET inhibitor), selumetinib (MEK inhibitor), adavosertib (WEE1 inhibitor) and vistusertib (TORC inhibitor). The primary endpoint of the study was the overall response rate (ORR), considered effective in almost all sub-study if ≥50%, assuming an ORR of 20% for single agent paclitaxel in the same setting. Excluding the arms closed due to early termination of drug development or lack of efficacy, the ORR observed ranged from 50% (10/20, 95% CI: 28.0–71.9) of savolitinib monotherapy (arm 4) to 24% (6/25, 95% CI: 7.3–40.7) of adavosertib/paclitaxel combination (arm 2). VIKTORY was one of the first umbrella trials specifically designed for gastric cancer patients, and allowed treatment of many patients with a biomarker-matched therapy, with encouraging response rates. Other examples of umbrella designed protocols are ALCHEMIST (NCT02194738), whose updated enrollment status has been recently reported [14], and Lung-MAP (NCT02154490) for lung cancer [15,16], ADAPT (NCT01781338) and plasmaMATCH (NCT03182634) for breast cancer [17,18].

## 3. Basket Trials

Basket trials are prospective clinical studies testing one or more targeted agents in a population of patients selected by matched biomarker(s) [19] (Figure 1b). In contrast with umbrella trials, in a basket trial subjects are not selected by histology (histology-independent screening), including patients affected by different types of cancer [20]. Thus, in a basket trial, a common screening procedure is required for patients with different histologies and eligibility is related to the presence of the same mutational or genomic characteristics, or of different predictive biomarkers targeted by the same compound [21].

Entrectinib, a TRK A/B/C inhibitor, has been investigated in three phase 1/2 basket studies (ALKA, STARTRK-1, STARTRK-2; EudraCT 2012-000148-88; NCT02097810; NCT02568267) in patients with metastatic or locally advanced NTRK fusion-positive solid tumours [22]. The integrated prespecified analysis of these trials included 54 patients with 19 distinct histologies, and many patients with rare tumor types were represented as sarcoma (24%) and mammary analogue secretory carcinoma of the salivary gland (13%) [23]. This is an example of inclusive and histology-independent design, which could also offer a potential biomarker-based therapy to patients with highly rare cancers. Another example of basket study is the French SHIVA trial. This was a proof-of-concept, phase 2 trial of biomarker-matched targeted agents in patients with refractory cancers. Primary endpoint was progression-free survival (PFS). Participants underwent next generation sequencing (NGS) and immunohistochemistry analyses, and were further assigned to receive matched targeted treatment (*n* = 99) or treatment at physician’s choice (*n* = 96). Median PFS was 2.3 months (95% CI 1.7–3.8) in the experimental group versus 2.0 months (1.8–2.1) in the control group (HR 0.88, 95% CI 0.65–1.19, *p* = 0.41) [24]. Despite the negative result, SHIVA represented a model for a histology-agnostic, controlled and randomized trial.

## 4. Platform Trials

**Figure 2:**
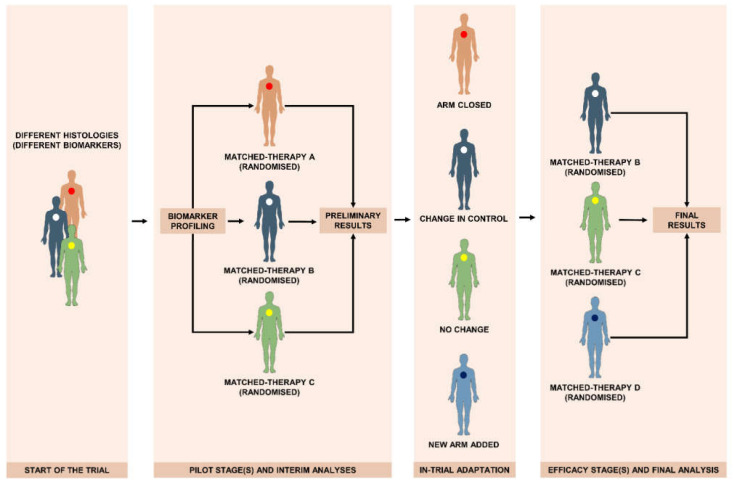
Platform trial design: main phases and data flow.

Platform design consists of a common randomized trial structure, to study multiple targeted therapies in multiple biomarker-selected populations [25] (Figure 2). The main characteristic of platform trials is a “fluid” structure, where continuously therapeutic arms and/or sub-studies enter and drop from the original trial design (or platform) on the basis of a decision algorithm [26]. These trials are also described as “adaptive”, referring to a within-trial adaptation where assignment probability to specific treatment arms is modified according to prespecified success of failure thresholds, to allow patients to receive interventions that perform most favorably [25,27].

STAMPEDE (Systemic Therapy for Advancing or Metastatic Prostate Cancer) represents a flagship randomized platform trial with a multi-arm multi-stage (MAMS) design, which allows several treatments to be assessed concurrently with preplanned interim adaptations [28]. The trial, at its initial pilot stage, was structured as a controlled six-arm, five-stage trial of different therapies for men with prostate cancer starting hormone therapy; it opened to accrual in 2005, and is still ongoing. Patients were randomly assigned to either the control arm or one of five experimental arms. Data on the “original comparisons” evaluating zoledronic acid, docetaxel and celecoxib (alone or in combination) have been already reported [29], and from 2005, the trial was modified multiple times, adding treatment arms with abiraterone and enzalutamide (alone or in combination) and radiotherapy [30,31,32]. The STAMPEDE trial is currently at the 21th version of the protocol, with the aim to study the activity of metformin and transdermal estradiol [33].

After the STAMPEDE experience, other adaptive platform trials started for different tumor types. FOCUS-4 was a biomarker-stratified trial program in metastatic colorectal cancer consisting of parallel molecularly stratified and randomized comparisons of maintenance therapies after first line chemotherapy. At the first preplanned interim analysis (March 2016), the FOCUS4-D sub-study, testing the anti-EGFR/HER2/HER3 tyrosine kinase inhibitor AZD8931 versus placebo in the all wild-type cohort, closed due to a lack of efficacy [34]. Moreover, recently the trial has been completed after the interruption of recruitment related to the COVID-19 pandemic in March 2020, and it closed the follow-up of all patients in October 2020 [35]. I-SPY 2 is an adaptive clinical trial platform designed to improve outcomes in high-risk breast cancer patients, testing new drugs in neoadjuvant settings [36]. Patients with early-stage breast cancer are categorized into molecular subgroups on the basis of hormone receptor status, HER2 status and risk according to a 70-gene assay (the MammaPrint scores [MammaPrint, Agendia] [37]. These groups are adaptively randomized to control arm or experimental arms. The trial is currently ongoing, and a total of 17 agents or combinations entered into the I-SPY 2 platform so far [38]. Recently, results of the arm investigating clinical activity of the combination of the anti-PD-L1 durvalumab, the PARP inhibitor olaparib and paclitaxel (compared with paclitaxel alone) have been reported [39].

## 5. Opportunities and Limitations of Biomarker-Driven Master Protocols

Basket and umbrella trials are characterized by a histology-independent and -dependent design, respectively. Instead, the label of “platform trial” is not “histology-based”, but refers specifically to the adaptive nature of the study design. This type of trial over-structure could comprise single or multiple cancer types (and single or multiple biomarkers and matched-therapies). Thus, many umbrella trials that have also an adaptive design, could be also considered platform trials (Lung-MAP, ADAPT, I-SPY-2, ALCHEMIST), often leading to mislabeling and overlapping definitions.

Master protocols, and particularly the studies with a platform design, try to overcome common limitations of classical randomized clinical trials (RCT) [11,40]. This adaptive and “fluid” design allows to abandon the traditional approach of phase 2 trial as a learning stage, followed by the confirmatory phase 3 study. Many umbrella/platform trials are defined as “seamless” phase 2/3 trials: the same study proceeds across the phase 2 and 3 with a go/no-go decision based on interim (futility) analyses [41]. This approach could accelerate drug development with a reduction of regulatory stand-by times between phases 2 and 3, preventing the exposure of a larger number of subjects to potential harmful and useless treatments in the phase 2–3 switch [42]. On the other hand, it must be considered that the phase 2 and phase 3 part of a seamless trial could not have the same characteristics in terms of design and statistical plan. This issue has been extensively reported for the seamless phase 1/2 trial, but could be applied at any phases [43]. In particular, baseline patients’ characteristics could differ through the two parts of the study (as different population could be included) and the randomization process could be present in none, one or both parts of the trial. When study population differs from phase 2 and phase 3 part of the trial, data analysis should carefully adjust for patients’ characteristics. As stated, master protocols give the opportunity to speed up screening and enrollment, especially in rare cancer settings (basket trials) or when multiple biomarkers (and matched therapies) could be tested in a single histology (umbrella trials). The dynamism of the platform design could also reduce the cost- and time-wasting procedures of design, and conduct subsequent single, independent RCTs when new evidences accumulate in the same setting. Notwithstanding when clinical trials are focused on a biomarker, some limitations could emerge. The first issue regards the screening process: especially in the umbrella trials, molecular and genomic profiling is often centralized, in order to allow testing multiple predictive alterations at the same time and with the same method [44]. This facilitates the process when high-quality (and -amount) of tissue is available (e.g., breast and colon cancer), but could slow profiling procedures in cancer types with known difficulties of tissue sampling (e.g., lung cancer [45]). On the other hand, when only one biomarker should be assessed to access to the study (e.g., basket trials), investigator-assessed profiling could be preferred. The risk, in this case, is that different profiling methodology could be used, leading to a lack of harmonization of the screening procedure and to the difficult generalizability of the results. The background of a basket trial is often based on the extrapolation of the clinical effect of a compound from a single biomarker-matched population, and on the hypothesis of a similar activity in others. If this characteristic has the advantage to allow the enrollment of different (and rare) cancer types, it should be addressed with caution, considering that whilst expressing the same biomarker, different tumor types may not respond equally to the same targeted therapy. In the phase 2 basket trial of vemurafenib in BRAF V600-mutated cancers, clinical activity was observed in several tumor types, including non-small cell lung cancer (NSCLC) and three rare histologies: Erdheim–Chester disease; Langerhans’-cell histiocytosis; and anaplastic pleomorphic xanthoastrocytoma. Nevertheless, in the cohort of patients with colorectal cancer who received vemurafenib monotherapy, no responses were observed [46]. This underlines how different tumor types with the same molecular biomarker might differ in their sensitivity to the same targeted agent. So, basket studies are considered discovery-based trials that generally occur early in drug development, assessing preliminary activity on matched-therapies that often need further investigation [47].

Both basket and umbrella trials could have one (or more) control arms. The choice of a control arm derives from previous clinical evidences, and from the availability of active alternatives in the same setting. The histology-dependent design of umbrella trials allows to add the same control arm in all the sub-studies, while in basket trials, many different control arms should be chosen as much as how many cancer types are included. As an alternative, the control could be the “standard-of-care” per investigators choice, different for any histology (as in the SHIVA trial). However, when no active therapeutic opportunities exist, both study types allow also a placebo-controlled design. Differently, in platform trials that are typically randomized, control arms vary during the study, adaptively, as new evidence accumulates within and outside the trial. These characteristics have led to the development of many basket trials as proof-of-concept, single arm, not controlled studies, while many umbrella and platform trials span from exploratory, proof-of-concept applications to confirmatory ones [19].

Notwithstanding this, results of all these studies should be addressed with caution. As stated, especially exploratory trials (e.g., basket studies) could lead to misinterpretation of the real impact of biomarker-driven therapies. Thus, effectiveness of treatments approved on the basis of proof-of-concept trial (often under accelerated approval) or with proven efficacy in early phase setting only may need appropriate confirmation by post-licensing or late-phase studies [44].

## 6. Statistical Considerations

Master protocols are also characterized by advantages and issues in terms of statistical analyses. Despite these considerations being outside of the purpose of this article, some of them have to be briefly mentioned. Considering the biomarker-driven match, the awaited benefit in these trials is generally high, leading to a smaller sample size required if compared with a study without biomarker selection [48]. In addition, when a seamless phase 2/3 design is applied, sample size could further be reduced, since phase 2 portion of data can be incorporated to those of phase 3 (final) analysis [42]. In this case, characteristics of data from the phase 2 part of the study that are supposed to be used in the final analysis should be addressed prior to any interim data analysis, and specified in the trial protocol [43].

Nevertheless, in umbrella and platform trials, the sample size is generally calculated for every single sub-study, while in basket trials it could be calculated separately for each histology-dependent cohort (as in the vemurafenib basket trial) or for the entire study population, hypothesizing a common “effect size” (as in STARTRK-2 and SHIVA trial).

Master protocols, as stated, are designed to efficiently respond to multiple questions. However, the multiplicity of comparisons within a single dataset, as well as the number of subgroup to analyze, put at risk of uncontrolled type-I error (false positive results), leading to the need of appropriate statistical tools to minimize this risk [49,50]. Another major issue in master protocol planning is the choice of endpoints that reflect the previous considerations on the overall differences between the three design structures. In basket trials, the primary endpoint is often the response rate, as in umbrella and platform trials, it could change on the bases of the design of the single sub-study: from early endpoints as in the VIKTORY trial (ORR) to more robust and validated endpoints typically used in the large phase 3 trial (PFS, overall survival [OS]), as in some sub-studies of Lung-MAP and FOCUS-4. Nevertheless, the choice of endpoints that do not require years to be measured (as a response rate), even if they allow investigators to access results earlier, exposes the risk of abuse of surrogate endpoints, which many times could disturb their interpretation [51,52]. To avoid this, many master protocols are designed with early endpoints for the interim (futility) analyses and with more validated efficacy or survival endpoints for the final analysis. The STAMPEDE trial, for example, even at its beginning, comprised five stages with different endpoints: a pilot (early-phase) stage; three intermediate activity stages; and a final efficacy stage. The primary endpoints of these stages reflected the single sub-study (or stage) design and were safety, failure-free survival and OS, respectively. Anyway, it must be considered that endpoints used for futility analyses could vary, influencing early study termination and the overall trial structure (in the case of platform trials). As demonstrated by Goldman and colleagues, when treatment has no effect on both a surrogate endpoint and a more validate (survival) endpoint, the use of the surrogate one for futility analysis results in a substantial increase in early stoppages [53]. This conservative approach reduces the expected accrual and the duration of the study, which is required to assess the lack of treatment benefit (when this hypothesis is true). At the same time, it exposes to the risk of unjustified early stoppage when the effect is different on surrogate and validate endpoints. In particular, the dynamic, fluid design of the platform and seamless trials multiplies this risk, which should be considered during trial planning.

## 7. Conclusions

Master protocols, despite their intrinsic limitations and the difficulties related to their planning, coordination and conduction, represent a great opportunity in biomarker-driven clinical research to accelerate drug development in “precision oncology”. At the same time, these studies do not completely replace “classical” drug approval RCTs in terms of efficacy and safety level of evidence. In particular, this limitation is true for basket and other proof-of-concept studies, while platform trials could be considered the most efficient design to definitively overcome these issues in the future. The appropriate use of endpoints for interim and final analysis, focusing on survival and quality of life, the inclusion of randomization and control arms, should be preferred when feasible.

Notwithstanding this, the increasing complexity of master protocols data analysis and interpretation risks slowing the correct translation of knowledge in everyday practice if it is not linked to an adequate implementation of oncologist and methodologist training.

Continuous efforts should be made by companies, regulatory authorities and academic centers to maximize the planning of new master protocols and the correct conduction of ongoing studies.

## Data Availability

Not applicable.
